# Causal effects in microbiomes using interventional calculus

**DOI:** 10.1038/s41598-021-84905-3

**Published:** 2021-03-11

**Authors:** Musfiqur Sazal, Vitalii Stebliankin, Kalai Mathee, Changwon Yoo, Giri Narasimhan

**Affiliations:** 1 Bioinformatics Research Group (BioRG), Florida International University, Miami, 33199 USA; 2 Herbert Wertheim College of Medicine, Florida International University, Miami, 33199 USA; 3 Biomolecular Sciences Institute, Florida International University, Miami, 33199 USA; 4 Department of Biostatistics, Florida International University, Miami, 33199 USA

**Keywords:** Computational biology and bioinformatics, Systems biology, Mathematics and computing, Computational science

## Abstract

Causal inference in biomedical research allows us to shift the paradigm from investigating associational relationships to causal ones. Inferring causal relationships can help in understanding the inner workings of biological processes. Association patterns can be coincidental and may lead to wrong conclusions about causality in complex systems. Microbiomes are highly complex, diverse, and dynamic environments. Microbes are key players in human health and disease. Hence knowledge of critical causal relationships among the entities in a microbiome, and the impact of internal and external factors on microbial abundance and their interactions are essential for understanding disease mechanisms and making appropriate treatment recommendations. In this paper, we employ causal inference techniques to understand causal relationships between various entities in a microbiome, and to use the resulting causal network to make useful computations. We introduce a novel pipeline for microbiome analysis, which includes adding an *outcome* or “disease” variable, and then computing the causal network, referred to as a “disease network”, with the goal of identifying disease-relevant causal factors from the microbiome. Internventional techniques are then applied to the resulting network, allowing us to compute a measure called the *causal effect* of one or more microbial taxa on the outcome variable or the condition of interest. Finally, we propose a measure called *causal influence* that quantifies the total influence exerted by a microbial taxon on the rest of the microiome. Our pipeline is robust, sensitive, different from traditional approaches, and able to predict interventional effects without any controlled experiments. The pipeline can be used to identify potential eubiotic and dysbiotic microbial taxa in a microbiome. We validate our results using synthetic data sets and using results on real data sets that were previously published.

## Introduction

This term *microbiome* refers to a microbial habitat, and includes the microorganisms (bacteria, archaea, microbial eurkaryotes, and viruses), their genomes, and the surrounding environmental conditions^1^. The microbes in a microbiome are involved in complex, dynamic interactions among themselves as well as with the host environment. Balanced compositions and harmonious relationships in the microbiomes are associated with healthy environments. However, a *dysbiosis* (i.e., imbalance) can disrupt these relationships and are associated with human disease and environmental ills. A deeper understanding of microbial interactions within the microbiome is the overarching aim of our work. We hypothesize that many of the microbial relationships are a result of complex biological processes and are therefore causal in nature. While the etiology of a handful of infectious diseases can be traced back to a single species or strain of some pathogen, most diseases are complex and multifactorial. Uncovering causal relationships is thus an important first step toward understanding disease and also predicting the course of future treatments.

Great strides have also been made in *causal inferencing* from data. Starting from strong theoretical foundations, the notion of *conditional independence*^2^, the theory of *Bayesian networks*^3,4^, the notion of d-*separation*^5^, the development of efficient inferencing algorithms^6,7^, and the development of the *do-calculus*^8,9^, have made it possible to go from good experimental data sets to useful causal relationships and predictive capability for interventions.

With ground-breaking advancements in high-throughput sequencing technologies, it is now possible to examine microbial diversity in microbiomes with increased precision, and has led to a large number of research investigations on the associations between the microbiome and phenotypes such as obesity, neurological disorders, inflammation, immune disorders, metabolic diseases, and more^10–13^. Interest in constructing causal networks for microbiomes is recent^14,15^. Focused experiments in the laboratory to elicit causal relationships within microbiomes do exist^16,17^, but do not employ computational causal inferencing approaches. Sazal et al. were among the first to construct causal networks for microbiomes^18^. They have showed that directed edges in causal networks inferred from metagenomics data using the R-based tool bnlearn^19^ are consistent with known colonization order^20^. Kitsios et al. investigated data from 56 patients with bacterial pneumonia and constructed a network of relationships between microbial taxa and other clinical variables^21^. Although they used the web-based inferencing tool, *CausalMGM*^22^, to construct a probabilistic graphical model, their work falls short of doing causal inference and shows an undirected network of associational relationships for lung microbiomes. Mainali et al. used Granger causality to infer causality, but their work requires microbiome data from longitudinal studies^23^. Literature on interventional studies of microbiomes are limited to laboratory experiments. Causal impact on the gut microbiome by nutrients^24^ and diet^25,26^ have been studied.

A significant advantage of constructing causal networks is that it allow us to study *interventions*, thus making it possible to measure the impact of a hypothetical action, i.e., the effect of “doing/intervening”. It helps us to answer interventional questions of the type: “if a person consumes a specific antibiotic, how will the abundance of taxon *A* in his/her gut change?” or “what is the expected abundance of *B. longum* if the relative abundance of *C. difficile* is fixed at 0.1?” We apply the interventional do-calculus designed by Pearl and others^27^ to data from microbiome studies. In particular, we apply the techniques to reanalyze the extensive gut microbiome data available for *Inflammatory Bowel Disease* (IBD). Dysbiosis of the gut microbiome is associated with IBD, colorectal cancer, obesity, and much more. However, the relationships between microbial taxa are complex and the experiments required to understand the causal mechanisms are expensive and time-consuming, and therefore remain poorly understood. This work attempts to tease out some of these relationships.

While causal networks describe inferred causal relationships between entities, the question we ask is: *How to quantify the causal effect of one entity on another in a microbiome?* In this work, causal networks were constructed and intervention calculus was applied to the resulting network to estimate the pairwise causal effects of covariates on each other and on specific response (outcome) variables. A scoring method is proposed to measure *causal effects* between pairs of entities and the *causal influence* of individual entities on all others. By augmenting the data with disease information, we construct networks called *disease networks*, which were used to identify the taxa playing key roles in the healthy and disease states. The pairwise effects provides useful information on the magnitude of the interaction (direct or indirect) between two specific taxa. However, in the context of microbiomes, dysbiosis is a community phenomenon. A pathogen can impact the whole community, not just a specific taxon. The concept of *causal influence* is an attempt to quantify the contribution of a microbial taxon to the dysbiosis of the microbial community. Finally, the concept of *disease networks* provide a framework to quantify the causal effect of individual taxon on the disease variable (or a health outcome). In summary, the results presented here suggest a way to identify “dysbiotic” and “eubiotic” microbes in microbiomes.

The paper is organized as follows. Section “Algorithm” discusses the Algorithms used in this paper; the “Results” section includes the details about data, findings from the experiments, and validation; the “Discussion” section summarizes the conclusions from the analysis, the arguments about the hypothesis, future directions, and concluding remarks; the “Methods” section describes the problem formulation and how the algorithms are applied to reach conclusions; and finally the Data Availability section gives the source of the data used in this paper.

## Algorithms

In this paper we primarily used three algorithms: a modified version of *PC-stable* for constructing causal networks^28^, an interventional calculus method for computing causal effects^29^, and an algorithm to identify *Y-structures*^30^. All the abovementioned algorithms are discussed here briefly.

### Causal structure

A *Bayesian network* (BN) is defined as a directed acyclic graph (DAG), $G = (V,E)$, where the *n* vertices from *V* represent *n* random variables from the set $X = \{X_1, \ldots , X_n\}$, each with its own probability distribution. Also, the *m* directed edges in *E* represent probabilistic relationships between the variables from *X*. If variables $X_i$ and $X_j$ are either marginally or conditionally independent, then the construction of the BN eliminates the edge between $X_i$ and $X_j$. Consequently, the DAG *G* can be associated with *P*, a joint probability distribution factorized as shown in Eq. (1)^31^.

1 $$\begin{aligned} P(\mathbf{V} ) = \prod _{j=1}^{n}P(X_i | Pa(X_i)). \end{aligned}$$

A *causal structure* or *network* (CN) is a BN with additional properties and interpretations. In a CN, an edge $X_i \rightarrow X_j$ means that $X_i$ was inferred to have a direct causal effect on $X_j$, while the lack of edge between $X_i$ and $X_j$ means that they are either marginally or conditionally independent. As with BNs, it is possible to compute any marginal probability on the variables in *X* in a CN. However, the edges of a CN clearly have great significance from a causal perspective.

**Figure 1:**
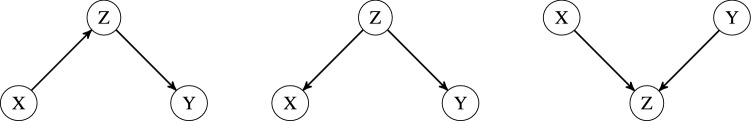
Left: causal chain, middle: fork or common effect, right: $v-$structure; from three variables *X*, *Y*, *Z*.

In the *causal structure*, the DAG encodes conditional dependence and independence relationships in the edges and in the joint probability function *P*. We discuss three important local substructures within causal structures that impact the independence relationships, and these include: *chains*, *forks*, and *v*-*structures* as shown in Fig. 1. In a chain, *X* and *Y* are connected by a directed path through node *Z*. The important consequence of a chain is that if no other paths exist from *X* to *Y*, then the two variables *X* and *Y* are conditionally independent given the intermediate *Z*. Note that the above property would hold even if *Z* is a set of nodes that intercepts every chain from *X* to *Y*. In a fork, variable *Z* is a “common cause” for variables *X* and *Y*. An important consequence of a fork is that if there are no directed paths between *X* and *Y*, then they are independent conditional on *Z*. Again *Z* could be a set of nodes that commonly cause *X* and *Y*. Finally, set *Z* is a “collider” node between *X* and *Y*, if it is the “common-effect” forming a *v*-structure (also called inverted fork). An important consequence of the *v*-structure is that if *X* and *Y* are unconditionally independent, then they become dependent when conditioned on *Z* and the descendants of *Z*.

Several DAGs can encode the exact same joint probability function. These DAGs are called *Markov* equivalent networks. Such DAGs form a *Markov* equivalence class and can be uniquely represented by a *CPDAG*, with the same skeleton and *v-structures*. CPDAGs allow both directed ($\rightarrow$) and undirected (−) edges. CPDAGs are specialized causal structures, but allow interventional calculus to measure causal effects.

### Construction of causal networks

To construct the CNs^28^, the network constructed by *PC-stable* algorithm was enhanced by incorporating correlational patterns (sign of correlation coefficients) on the edges and that help us to interpret the results biologically. The main steps of *PC-stable* algorithm we used to construct causal networks are as follows. 
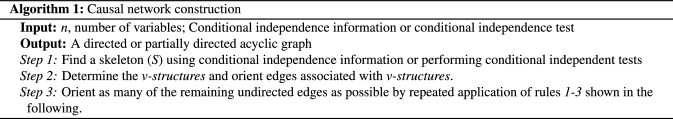


In *Step 1*, the algorithm starts with a complete undirected graph and then performs a series of conditional independence tests to eliminate as many edges as possible. The remaining undirected graph is referred to as the *skeleton*.

*Step 2* is key to inferring a causal structure, and uses the concept of *v*-structures, which are defined as follows. For any three nodes representing variables $X_i,X_j,X_k$ in a skeleton *S*, if $\{X_i,X_j\}$ and $\{X_j, X_k\}$ are edges in *S*, but $\{X_i, X_k\}$ is not, and if edges are oriented as $X_i \rightarrow X_j \leftarrow X_k$ then the triple $(X_i,X_j,X_k)$ is called a *v*-structure. Triples satisfying the *v*-structure property can be identified in the skeletons using conditional dependency tests, following which edges are appropriately directed to form a *v*-structure. The variable $X_j$ in the triple forming the *v*-structure represents a “common effect” of $X_i$ and $X_k$. These *v*-structures are critical in assigning directions to some of the edges of the skeleton.

In *Step 3*, three rules^28^ are applied repeatedly to orient edges not already in *v*-structures.

**Rule 1:** Orient $X_j - X_k$ as $X_j \rightarrow X_k$ whenever (a) there is a directed edge $X_i \rightarrow X_j$ and (b) $X_i$ and $X_k$ are not adjacent.
**Rule 2:** Orient $X_j - X_k$ as $X_j \rightarrow X_k$ whenever there is a chain $X_j \rightarrow X_i \rightarrow X_k$.
**Rule 3:** Orient $X_j - X_k$ as $X_j \rightarrow X_k$ whenever there are two chains $X_j - X_i \rightarrow X_k$ and $X_j - X_l \rightarrow X_k$ given that $X_i$ and $X_l$ are not adjacent.

### Intervention

**Figure 2:**
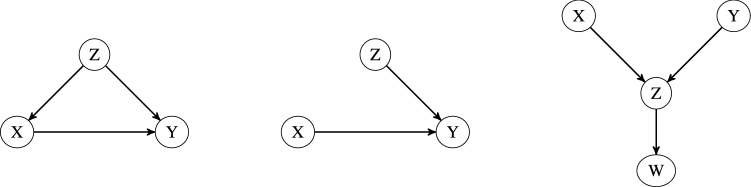
Left: A DAG (G) representing relationship among random variables *X*, *Y*, *Z*; middle: a mutilated network ($G_m$) representing intervention on *X*; yight: Y-structure from four variables *X*, *Y*, *Z*, *W*.

In the context of causality there are two types of data: *observational* and *interventional*. Observational data arise from observational experiments, not to be confused with randomized controlled experiments. On the other hand, interventional data are recorded after perturbations using external agents. Interventional queries can be answered using interventional data (also called *experimental data*), where some variables in the system are set/held to a fixed value by an external agent. However, interventional data can only answer queries when the variables are set to the specific value used in the experiment. A general need is to answer queries when the variables are set to *arbitrary* values for which experiments were not carried out. The challenge is to infer *causal relationships*, infer the result of arbitrary interventions, and to infer the magnitudes of causal relationships only from observational data. A *mutilation* operation at a node *X* in a DAG is obtained by deleting all incoming edges into *X*. A *mutilated network* with respect to node *X* in a DAG is derived from the original network by performing a mutilation operation at *X*. Figure 2 shows a network (left) and the mutilated network (middle) obtained by a mutilation operation at node *X*.

Intervention is expressed using Pearl’s $\mathbf {do()}$ operator^32^. $P(Y| \mathbf {do (X=a)})$ denotes the distribution of *Y* if the value of *X* is set to *a*. The post-interventional densities are expresses using the formula in Eq. (2)^33^.

2 $$\begin{aligned} P(\mathbf {V} | \mathbf {d o(x)})=\left\{ \begin{array}{ll}\prod _{V_{i} \in \mathbf {V} \backslash \mathbf {X}} P\left( V_{i} | Pa\left( V_{i}\right) \right) , &{} \text{ if } \mathbf {X}=\mathbf {x} \\ 0, &{} \text{ otherwise } \end{array}\right. \end{aligned}$$

Note that a controlled experiment can potentially answer interventional questions, but may be either prohibitively expensive, impossible to execute, or unethical to perform. Causal calculus allows us to answer such interventional questions using purely in silico methods. We clarify that data collected from research studies (e.g., a microbiome study) are considered as *observational* data, and not the result of *controlled interventions*, which require that variables be artificially held at specific values. Conditional expectation is given by $E[Y|X=x]$, while interventional expectation is given by $E[Y|\mathbf {do}(X=x)]$, which is the expectation of *Y* if every sample in the population had variable *X* fixed at value *x*^34^. Observational probability *P*(*y*|*x*) is thus different from interventional probability $P(y|\mathbf {do}(x))$. Observational distribution *P*(*Y*|*x*) describes the distribution of *Y* given that the observed value of variable *X* is *x*. On the other hand, interventional distribution of *Y* is the distribution if we set the variable *X* of all samples to take value *x*, while other variables are held unchanged^35^. To achieve $\mathbf {do}(X = a)$, we delete all incoming edges to node *X*, fix its value at *a*, and then perform the necessary computations on the resulting network (Figure 2 shows an original network and corresponding mutilated network).

### Interventional calculus

A causal model has both probabilistic and causal interpretations. From a probabilistic perspective, as mentioned earlier, each variable $X_i \in \mathbf{X}$, is independent of all its non-descendants when conditioned on its parents, $Pa(X_i)$, a condition called the *Markov condition*. From a causal perspective, a directed edge $(X_i, X_j)$ in *G* represents a direct causal impact exerted by $X_i$ on $X_j$^36^. The left side of Eq. (2) is the post-interventional distribution of *G*, while the right side is the pre-interventional distribution from the mutilated graph, $G_m$. To study the magnitude of the causal effect of $X_i$ on $X_n$, where $i \ne n$, we make $X_n$ the outcome variable and apply standard computations. The distribution of $X_n$ after an intervention $\mathbf {do} (X_i = x_i)$ can be estimated by integrating over all variables corresponding to $Pa(X_i)$. Assume that $X_i$ has at least one parent, i.e., $Pa(X_i) \ne \emptyset$, and that $X_n \notin Pa(X_i)$. Note that if $X_n \in Pa(X_i)$, then $P(X_n | \mathbf {do})X_i=x_i)) = P(X_n)$ because the causal network *G* is acyclic. Thus, if $X_n \notin Pa(X_i)$, then

3 $$\begin{aligned} P(X_n | \mathbf {do}(X_i=x_i)) = \idotsint _{Pa(X_i)} P (X_n | X_i = x_i, Pa(X_i)) P({Pa(X_i)}) d({Pa(X_i)}), \end{aligned}$$

where $P(Pa(X_i))$ is the joint distribution of the parents of $X_i$, and the integral is over all possible values that can be taken by the parents of $X_i$. Taking expectation on both sides, and assuming $X_n \notin Pa(X_i)$, gives us the following:

4 $$\begin{aligned} \mathbb {E}[X_n | \mathbf {do}(X_{i}=x_i)] = {\displaystyle \idotsint _{Y_i} \mathbb {E}(X_n | x_i, Y_j) P(Y_j) d(Y_j),} \end{aligned}$$

where $$Pa(X_i) = \{Y_1, \ldots , Y_p\}$$.

### Causal effect and causal influence

The magnitude of causal effect of $X_i$ on $X_n$, upon the action $\mathbf {do}(X_i = x_i)$ is denoted by $C(X_i, X_n)$ and is given by:

5 $$\begin{aligned} C(X_i, X_n)=\frac{\partial }{\partial x} \mathbb {E}[X_n | \mathbf {do}(X_i=x_i)]. \end{aligned}$$

If we assume that the joint distribution of *n* random variables $X_1, \ldots , X_n$ (as expressed in Eq. 1) is Gaussian/normal, then the causal effect values of $X_i$ on $X_n$ as described in Eq. (5) can be computed using linear regression because the normality implies that $\mathbb {E}(X_n| Pa(X_i), X_i=x)$ is linear in $x_i$ and $Y_j \in Pa(X_i), j = 1, \ldots , p$, as shown below^27^:

6 $$\begin{aligned} E\left( X_n |Y_1,\ldots ,Y_p,x_i \right) =\alpha +\gamma x_i+\sum _{j=1}^p \beta _{j}^{T} Y_j, \end{aligned}$$

for some values $\alpha , \gamma \in \mathbb {R}$ and $\beta \in \mathbb {R}^p$ represents a vector of regression coefficients of the parents of $X_i$. Thus, as shown in^29^, the magnitude of **causal effect** of $X_i$ on $X_j$ is given by:

7 $$\begin{aligned} C(X_i, X_n)= \gamma , \end{aligned}$$

where $\gamma$ is as dictated by Eq. (6). Note that,the linear regression model is only applied in the quantification step, which comes after the structure learning step, i.e., after the structure of the DAG or partial DAG representing the causal structures (qualitative relations) are inferred. At that time, the regression is only applied to connect the distribution of a random variable with that of its immediate parents in the causal structure. Thus, by the time, regression is applied, the nodes/variable involved in the relationships are already inferred.

The notion of the quantity, *causal effect*, defined above is a pairwise measure of how much one variable causally impacts another. Here we define another quantity called the **causal influence** of a node in a causal network, defined as the sum total of absolute value of the causal effect it exerts on every other node. Let $T = \{B_1, B_2, \ldots , B_n\}$ be the set of nodes representing random variables.

Equation (5) gives the causal effect of $B_i$ on $B_j$. The *causal influence* of node $B_i$ is given by the quantity:

8 $$\begin{aligned} CI(B_i) = \sum _{j \ne i} |C(B_i, B_j)|. \end{aligned}$$

Since causal effect values can take negative values as well, the formula for causal influence involves the sum of the absolute values. This prevents individual causal effect values of highly influential nodes from canceling each other out. To avoid confusion, we note that the definition of causal influence is the sum of the causal effect of the taxon on every other taxon, regardless of whether the corresponding nodes have a direct causal link or not. This ensures that we also attribute to the causal influence of a node, all effects that it might have indirectly.

### Y-structures

A *v*-*structure* over variables *X*, *Y*, *Z* is shown in Fig. 1. There are two directed edges $X \rightarrow Z$, $Y \rightarrow Z$ and there is no edge between *X* and *Y*. However, the *v*-*structure* is not enough for discovering that variables *X* or *Y* causes *Z* without the assumption that the structure is causally sufficient.

The concept of *Y*-*structures* is an extension of the concept of *v*-*structures*. As shown in Fig. 2 (right), a Y-structure contains four nodes ($\{W, X, Y, Z\}$), with 3 of the 4 vertices forming a V-structure ($\{X, Y, Z\}$). If there is an edge from *Z*, the center of the V-structure, to the node *W*, and if there are no edges from *X* to *W* or from *Y* to *W*, then the nodes *X*, *Y*, *Z*, *W* form a *Y*-*structure* in the causal network. We will refer to the edge directed from *Z* to *W* as the *Y-leg*. Theoretically, we know that if a *Y-structure* is learned from data, the Y-leg represents an unconfounded causal relationship^30^, making the Y-leg edges valuable for biological interpretations.

## Results

### Synthetic data

Since networks with known causal relationships are not readily available, we first performed experiments with synthetically generated data sets. We generated random networks with variable number of nodes ($n = 9, 17, 26, 35$) with different number of edges. For each random network (ground truth), we generated $m = 1000$ samples, and then attempted to see (a) if the network that generated the data could be recovered using our inferencing tools, and (b) if the causal influence values match the values computed from the ground truth network. The procedure for generating the synthetic networks and the corresponding data set is as follows. We generated random *DAG*s with predefined number of nodes and edges using *pcalg* package^29^. Finally we generated a specified number of (random) samples from the synthetically generated DAG using a logic sampling algorithm^37^.

The summary statistics of inferred networks from the synthetic data are shown in Table  1. We report precision, recall, F-1 score, and accuracy. The true positive (TP) rate is defined as the number of correctly inferred directed edges in the inferred network with respect to the true network. This above performance metrics were averaged over 100 experiments. A false positive (FP) rate is defined as the number of directed edges not present in the true network, but present in the inferred network. False negative (FN) rate is defined as the number of directed edges present in the true network, but not in the inferred network.

**Table 1:** Network configuration (number of nodes, directed edges), the number of directed edges in each synthetic true network, precision, recall, F-1 score, and accuracy.

Network size, *n*	# of directed edges, *e*	Precision	Recall	F-1 score	Accuracy (%)
9	10	0.95	0.88	0.92	85.00
17	33	0.88	0.92	0.90	82.00
26	65	0.88	0.88	0.88	78.61
35	55	0.89	0.87	0.88	78.36

**Figure 3:**
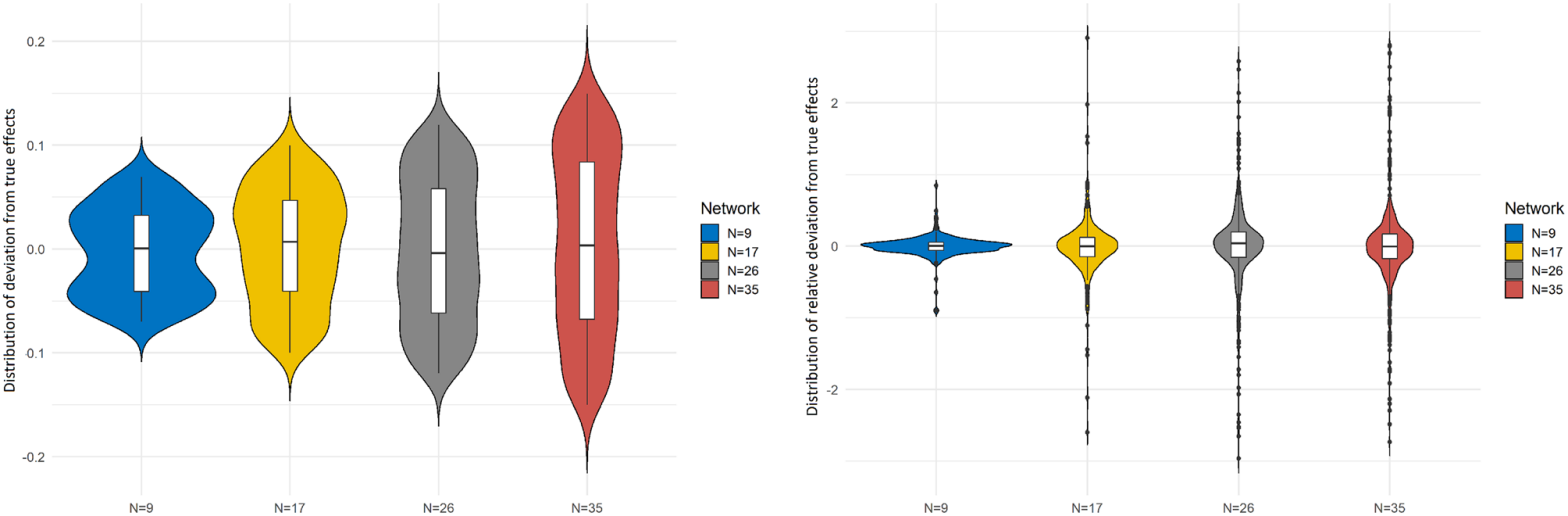
Distribution of the deviation of estimated causal effects from true causal effects (i.e., true value–estimated value) for all four sets of synthetic networks (left); distribution of the relative deviation of estimated causal effects from true causal effects, i.e., (true–estimated)/true from the experiments with all four sets of synthetic networks (right). This figure was generated using the R package, ggpubr^38^.

For each case, we learned the causal network and computed the causal effects between every pair of nodes. We also computed the deviation of estimated effects from the true effects, measured as $true - estimated$. Similarly we computed the relative deviation of estimated effects from the true effects, measured as $(true - estimated)/true$. The distribution of deviation and relative deviation values are shown in Fig. 3 as violin plots.

**Figure 4:**
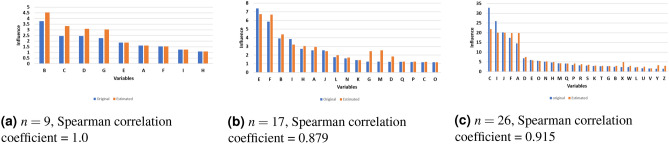
Pairwise comparisons between the true causal influences of individual nodes in three random synthetic networks with that of causal influences of the same nodes in inferred causal networks. The values are shown as paired bar charts ordered by decreasing true influence values. The ordering of the inferred influence for the top ten most influential variables is consistent with the true values.

Besides computing the pairwise causal effects we also calculated *causal influence* from the synthetic data. Figure 4 summarizes the comparison of influence values between true and inferred networks. Figure 4 compares the true causal influence values (computed from the ground truth CN) with the causal inference values from the inferred network. The figure shows that the causal influence values are reasonably close to the true values. This is seen by the difference between the bars in the bar charts. More importantly, it shows that even though the true values are sometimes different from the true values, the ordering of the nodes sorted by decreasing causal influence values is very close to the true values. In order to support a statement on the rankings, we applied *Spearman* correlation and showed that the correlation coefficients are high, thus showing that the sorted order of the two lists are remarkably consistent.

### Real data set

We constructed the causal networks from the data sets mentioned in Table 2 obtained from the IHMP study^39^. In each of the resulting causal structures, nodes represent random variables for one of two things—relative abundance of taxa, and disease status. In the visualized networks, the size of each node is proportional to the average value of that variable in the cohort. The color of each node represents the phylum to which the corresponding taxon belongs. Taxa from the the same phylum have the same color. Firmicutes taxa are colored with cyan, Bacteroidetes are colored blue, Proteobacteria are colored green, and Verrucomicrobia are colored purple.

**Table 2:** Three real data sets were used in this study.

Database: iHMP^39^		
Study	A. Diseased	B. Healthy
Ulcerative colitis (UC)	459	429
Crohn’s disease (CD)	749	429

We analyzed samples from Ulcerative colitis (UC), Crohn’s disease (CD), and healthy individuals (non-IBD). The number of samples from CD is relatively higher than the UC.

Edges represent the belief of direct causal relationships as inferred by the PC-stable algorithm. More importantly, the absence of an edge suggests that there is no direct causal relationship, although indirect relationships may exist. The color of the edges represents the sign of the correlation between the abundance vectors of the taxa represented by the nodes (green color stands for positive correlations, and red color for negative correlations). The transparency of each edge represents the confidence value for the predicted edge, computed by its bootstrap value. For each network, we estimated the confidence value of the predicted edges by computing the bootstrap value from 200 repetitions. An inferred causal structure may contain undirected edges if the data are not enough to support an edge orientation. Those undirected edges remain causally “uninterpretable”.

To quantify the statistical significance of the overall resulting causal structures we computed the maximum likelihood and log-likelihood scores of the networks we constructed. To obtain this measure for our networks, we randomly permuted the values in each row and created networks $N = 1000$ times and each time we calculated the log-likelihood. The fraction of the networks generated by random permutations whose likelihood is higher than that obtained for the predicted network is the p value or reported statistical significance value. The p values of the networks we used for analyses were less than 0.05.

Our experiments with the real data sets involved first inferring a causal network from the data and then computing all pairwise causal effect values. We created causal networks from the UC, CD, and non-IBD data sets separately using the PC-stable algorithm. *Outcome* causal networks (also called *disease causal networks* or simply disease networks) were also created by augmenting the data sets with a *disease* variable, corresponding to the categorical variable representing the disease status of the individual. Note that if disease severity were available for the subjects then this variable could also be continuous. Finally, we applied intervention techniques to measure causal effects and causal influence of each taxon.

### UC data set

**Figure 5:**
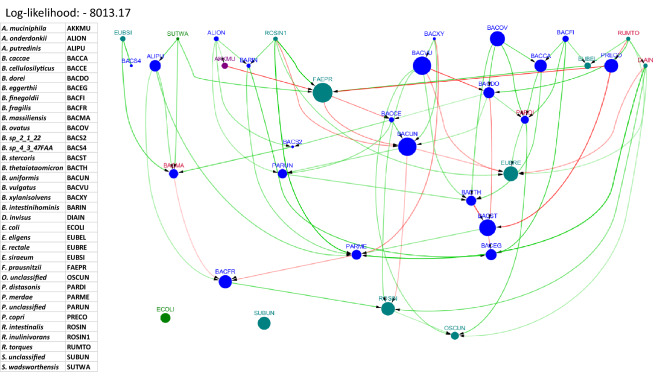
Causal network inferred from the data on subjects with UC from the iHMP data set. In this and all networks to follow, all directed edges point downward. The labels of potentially pathogenic bacteria are in red font. The log-likelihood score for the network along with a table of abbreviations used for the microbial taxa can be found to the left of the network. This figure was generated using the R package, bnlearn^31^, and visualized using Cytoscape 3.5.0^40^.

The causal network that resulted from the UC data set is shown in Fig. 5. Also we showed the causal network inferred from healthy cohorts in the supplementary (Figure S1).

**Figure 6:**
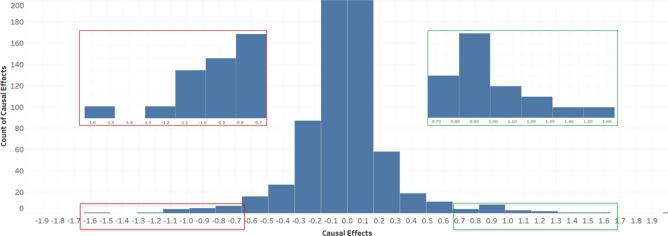
Histogram of pairwise causal effect values in the UC network. The top (green) and bottom (red) 15% are zoomed in for details.

The causal graph shown may be intuitive but is not easy to interpret precisely. In contrast, the intervention technique provides quantitative information that may lend itself more easily to interpretation. Thus, after creating causal networks, we computed causal effect values for all pairs of nodes, and *causal influence* values for all nodes. The distribution of pairwise causal effect values in the UC causal network is shown in Fig. 6. To identify the strongest pairwise causal relationships, we selected the top 15% (shown in green rectangle) and the bottom 15% (shown in red rectangle) to zoom in for further inspection.

**Figure 7:**
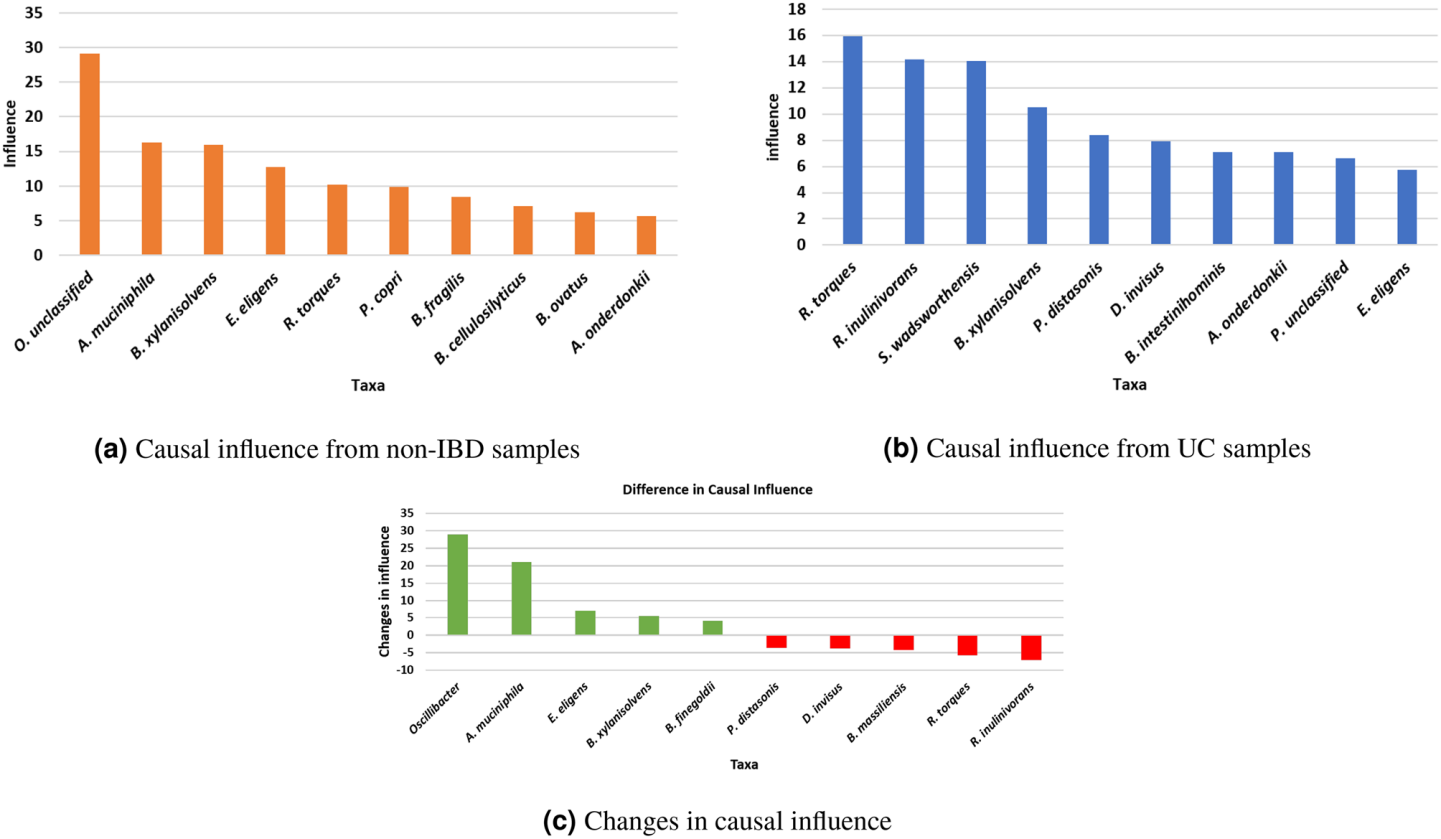
Top ten causally influential taxa from (**a**) non-IBD, (**b**) UC, and (**c**) the top 10 taxa with the highest change in causal influence from healthy to UC. Positive changes are shown as green bars and negative changes in red.

Next, we computed the causal influence measures for each microbial taxon (i.e., sum of absolute values of causal effects on every other variable). We then ranked the taxa as shown in Fig. 7a,b with the expectation that this list would highlight the most influential taxa in health or disease.

It also made sense to inspect the change in causal influence in going from the healthy cohort to the diseased cohort. If we denote $CI_H(i)$ and $CI_{UC}(i)$ to be *causal influence* of variable *i* in the causal network constructed from the healthy cohort and UC cohort, respectively, then $CI_H(i) - CI_{UC}(A)$ represents the change in influence for taxon *A*. Figure 7c shows the ten taxa with the highest change in causal influence. Green bars indicate higher causal influence values in healthy samples, while red bars indicate higher values in UC samples, suggesting that the taxa representing the green bars on the left of the chart are potentially eubiotic, while the taxa representing the red bars on the right of the chart play a dysbiotic role in subjects with UC.

#### Influence subnetworks in the causal network from UC data

Based on the *causal influence* values computed above, the top five taxa from the UC cohort were *R. torques* (RUMTO), *R. inulinivorans* (ROSIN), *S. wadsworthensis* (SUTWA), *B. xylanisolvens* (BACXY), *P. distasonis* (PARDI). We discuss our methodology to analyze their influence in greater detail. We include the detailed analyses of the sub-networks associated with *R. torques* (RUMTO) and *B. xylanisolvens*. Other subnetworks are discussed in the supplementary section.

We start with the most influential taxon, *R. torques*, labeled RUMTO in the UC network shown in Fig. 5. *R. torques* is a well known pathogenic taxon for UC. In the UC network, it has five outgoing directed edges connecting to *B. dorei*, *E. eligens*, *P. copri*, *E. rectale*, and *D. invisus*. Additionally, a total of 19 taxa (out of 35) are reachable by a directed path from *R. torques*. Further discussion on the analysis of the subnetworks can be found in the “Discussion” section. Similar discussion on the impact of *B. xylanisolvens* (labeled BACXY), another key player in UC pathogenesis can be found in the “Discussion” section.

**Figure 8:**
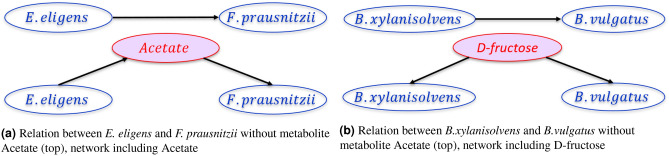
Unrolling causal relationships via metabolites. The presence of metabolites can make two causally dependent taxa conditionally independent.

To further investigate the fidelity of the causal network we dive deeper into some edges where mediator variables or metabolic data are available. As mentioned earlier *E. eligens* potentially interacts with *F. prausnitzii* via the metabolite, Acetate. When we included the concentration of Acetate from the associated metabolomics data into the analysis, the resulting network shows *E. eligens* to be independent of *F. prausnitzii* conditioned on Acetate concentration (see Fig. 8a). An investigation into the link from *B. xylanisolvens* to *B. vulgatus* in the UC causal network shows a similar behavior. Since both are known to be consumers of a metabolite named d-*fructose*, we created a causal network by including the concentration of d-*fructose* in the causal inferencing. As in the above example, *B. xylanisolvens* is independent of *B. vulgatus* when conditioned on d-fructose concentration (shown in Fig. 8b).

### Disease networks

Disease networks were created by combining data sets from one or more diseases (often including a data set from a healthy cohort) and producing networks with an additional node representing the outcome or *disease* status. For example in a disease network involving UC and healthy data sets, each sample from the UC cohort would have its disease variable set to 1 (0 for healthy samples).

**Figure 9:**
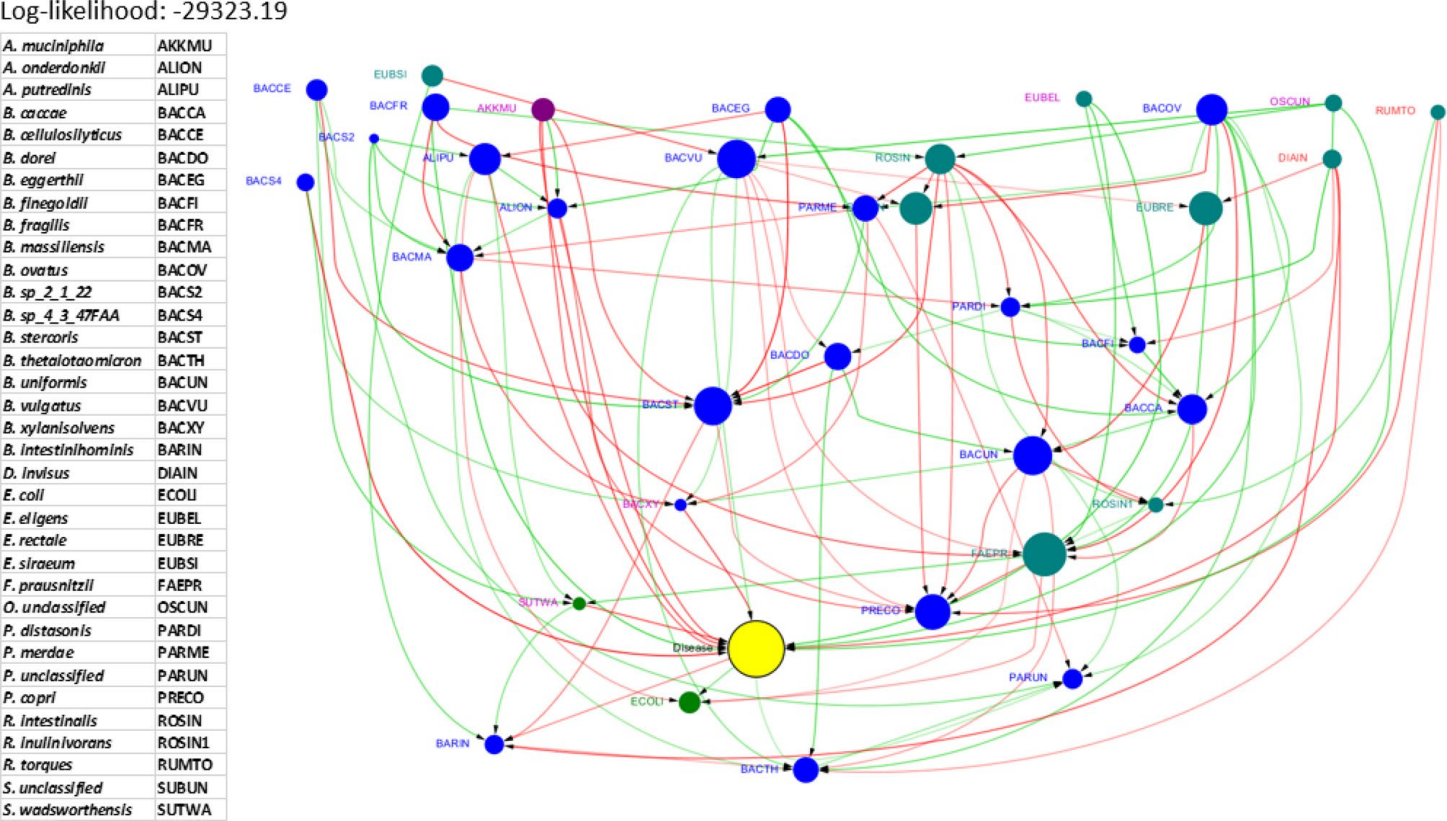
Causal network combining UC and non-IBD (healthy) data and introducing an additional *disease* node highlighted in yellow color. The labels of the known potential pathogens are in red color and the labels of the known potential beneficial bacteria are in purple color. This figure was generated using the R package, bnlearn^31^, and visualized using Cytoscape 3.5.0^40^.

Finally, we measured the causal effect of each taxon on the special *disease* node and sorted the list by their absolute value as shown in Table 3 for the UC disease network. We also reported the p-value of those pairwise effects of those from the bootstrapping with 100 repetition and same number of sample size.

**Table 3:** Sorted list of taxa (descending order) based on causal effects on *ulcerative colitis* “disease” node.

Cause	Effects on disease node	Potential behavior	p value
*Oscillibacter unclassified*	6.68	Beneficial	0.01
*Sutterella wadsworthensis*	6.34	Beneficial	0.03
*Eubacterium eligens*	5.29	Beneficial	0.10
*Bacteroides xylanisolvens*	4.73	Beneficial	0.06
*Alistipes ondedonkii*	4.68	?	0.09
*Bacteroides sp4*	4.04	?	0.04
*Dialister invisus*	2.53	Pathogenic	0.11
*Bacteroides ovatus*	2.38	Beneficial	0.08
*Ruminococcus torques*	1.41	Pathogenic	0.03
*Akkermansia muciniphila*	1.39	Beneficial	0.04

The p-value for each pairwise causal effect is also provided.

.

When we queried the published literature on this topic, we discovered that barring two, all the taxa listed in Table 3 were known to be either potentially pathogenic or beneficial, again supporting the claim that our approach helps to identify pathogenic and beneficial bacteria in healthy and diseased patients. Note that, we do not find enough evidence about the beneficial behavior or pathogenicity of *A. onderdonkii*, *B. intestinihominis* and their entries are marked with ? sign.

### Y-structure validation

In the *causal network* inferred from UC data set we have 18 *Y-structures*. We focus on the *Y-leg* edges from these Y-structures. Our experiments showed that the bootstrap values for the Y-leg edges with 100 repetitions for a given sample size ranged from 0.49 to 0.98. The mean, median, and standard deviation of bootstrap values were 0.68, 0.62, and 0.17, respectively. Theoretically, we know that the *Y-leg* edges cannot be confounded and bootstrap values also show high confidence on those edges. In other words, the endpoints of a *Y-leg* cannot have a “common cause”.

### Sensitivity analysis

For sensitivity analysis, we investigated the stability of the computed causal networks with perturbations in the input data. Ideally small changes in the input data should produce small or no changes in the model. The data sets were modified repeatedly as follows. For a randomly chosen sample, we generated a new random sample with same mean and standard deviation as the chosen sample. In this manner, we added $1\%, 2\%, 3\%, \ldots$ new samples to the data set until we the resulting input caused a significant change in the network structure. A significant change in the network is defined as the deletion of any edge from the original network with bootstrap value more than 0.50. For *UC* network, the first structural change to the network occurred after adding $7\%$ artificially vreated samples to the data set.

Similarly, randomly chosen samples from the input were deleted. Again, the first significant change in the network occurred after the deletion of $6\%$ of the samples. Thus, the computed causal networks are robust to an average of $6.5\%$ perturbations of samples. Similarly, the *Disease networks* are robust to perturbation of $8\%$ of the samples. One possible reason of less sensitivity of *Disease network* is that, *Disease network* is learned from larger number of data samples in comparison to *UC* network.

We also conducted a “substitution” experiment, which randomly perturbs the data across samples, neither deleting nor inserting rows in the data matrix. Again we started from $1\%$ and continue until we spotted at least one significant change. From the perturbation we found that the networks are more sensitive than deleting or adding samples. For *UC* network we encounter significant changes after randomly perturbing $4\%$ of samples and for the *Disease* network we noticed significant changes at $5\%$ of data perturbation. One of the possible reasons for the increase in the sensitivity is that, when we perturb data across samples, the relative abundance values are no longer coming from the same distribution and that makes the network more unstable.

## Discussion

Experiments with synthetically generated data sets (Fig. 4) shows that even though there are differences between true and inferred influence values, the relative ranking for most values remain consistent with that of true values. These experiments suggest that causal inference is a promising approach to analyzing microbiome data, especially when it comes to the identification of potentially dysbiotic or eubiotic microbes.

In the UC network (Fig. 5), *E. coli* and *S. unclassified* are isolated. It is known that *E. coli* is part of normal gut flora and evidence suggests that it is not playing a harmful role in the IBD gut^41^. The bacterial taxa *D. invisus*, *E. eligens*, *S. wadsworthensis*, *R. inulinivorans*, *A. muciniphila* are at the top of the network and have no incoming edges, suggesting that they exert an influence on most, if not all, of the descendant taxa in the lower part of the network. The highly abundant taxon *F. prausnitzii* from the Proteobacteria phylum has several incoming and outgoing edges, many colored red, suggesting that it has a strong negative influence on its descendant bacterial taxa and that its ancestors also impact it negatively.

The distribution of pairwise causal effect values in the UC causal network (see Fig. 6) is normally distributed with a peak at 0, suggesting that most pairwise causal effects are relatively small. The top 30% of the pairwise causal effects involve bacteria including *R. torques*, *F. prausnitzii*, *S. wadsworthensis*, *B. xylanisolvens*, *B. uniformis*, *P. copri*, all of which are known to be key players in UC pathogenesis.

Analysis of the data from non-IBD subjects (see supplementary Figure S1) shows the bacterial taxa *B. xylanisolvens, E. eligens, B. finegoldii, A. muciniphila* and some species of *Oscillobacter* to have the highest causal influence on the remaining taxa. These claims are supported in the literature, which show them to play a eubiotic role^42–45^. Analysis of the data from the diseased state (UC) shows that the taxa *R. torques, B. massiliensis, P. distasonis*, and *D. invisus* are the most influential. Again, the published literature supports the above claims by suggesting that these are potentially pathogenic^46–49^. Thus, we conclude that our methods allow us to identify potentially eubiotic and dysbiotic bacteria in cohorts of microbiome samples.

The subnetwork rooted at *R. torques* in the UC causal network (Fig. 5 shows a total of 19 taxa reachable from *R. torques*. Published work has suggested that *D. invisus* (DIAIN), a direct child of *R. torques*, is also associated with IBD^50^. Evidence also suggests that *R. torques* has an impact on pectin-modulated bacteria such as *P. copri* (PRECO)^51^. *R. torques* is also connected to *F. prausnitzii* (FAEPR) via *E. eligens* (EUBEL). It has been shown that *E. eligens* is a producer of acetate, which in turn is consumed by *F. prausnitzii*.

Causal influence values have already suggested that *B. xylanisolvens* (labeled BACXY) is a key player in UC. The analysis of the subnetwork rooted at *B. xylanisolvens*, which reaches 16 other taxa, is done in the context of metabolic networks from previously published literature. *B. xylanisolvens* is a producer of cellobiose, which may be consumed by *B. uniformis* (BACUN)^52,53^. *B. xylanisolvens* and *P. merdae* (PARME) both consume d-glucose^52,54^, making them potential competitors for glucose. This may explain the negatively correlated causal connection from *B. xylanisolvens* to *P. merdae*. *B. xylanisolvens* and *B. vulgatus* (BACVU) are both consumers of d-fructose^52,55^, making them potential competitors, although no evidence of competition is found in the network.

The analysis performed by selective addition of metabolite concentrations from associated metabolomic data (available from IHMP) was shown in Fig. 8a,b. This targeted analysis strongly suggests a role for the intermediate metabolites in the interaction between the pair of bacterial taxa mentioned. The claim is supported by the published literature on acetate and butyrate. After reaching the gut, carbohydrates resistant to digestion (commonly derived from dietary fibers) are degraded by gut microbiota to produce monosaccharides. These monosaccharides can be utilized by some bacteria including *E. eligens* in the gut to produce short-chain fatty acids such as acetate, butyrate, and propionate^56^. *Faecalibacterium prausnitzii* is a commonly known acetate consuming bacteria, it consumes acetate and produce various fatty acid including butyrate by utilizing glucose^57^. Interestingly, under *in vitro* conditions it was confirmed that the growth of *F. prausnitzii* is strongly stimulated in the presence of acetate^58^. *B.xylanisolvens* produce by-products such as acetate, succinate, and propionate. These fatty acids are the by-procducts of *xylose* and *sugar* fermentation. *B. xylanisolvens* is able to produce acid from many sugars such as glucose, mannitol, sucrose, glyercol, fructose, galactose, and melibiose^43,52^. Similarly, *Faecalibacterium prausnitzii* produces butyrate, formate, and lactate using fructose, oligofructose, and inulin^59^. Also, from the controlled experiment it is evident that treatment with fructans led to an increase of *F. prausnitzii*^60^. Due to the scarcity of data and knowledge-bases, many edges cannot be verified via metabolic networks. However, from the evidence it is understandable that metabolites play a huge role in the causal relationships in microbiomes.

Bacterial taxa that play an important role in the causal networks of healthy cohorts, but play a less influential role in the networks for disease cohorts are inferred as playing a eubiotic role within the microbiome. For example, *B. xylanisolvens, E. eligens, B. finegoldii, A. muciniphila* have the largest reduction in their causal influence values between the healthy and the diseased cohorts (Fig. 7) and their beneficial roles are confirmed by the literature^43,52,61–64^. Bacterial taxa that play an important role in the causal networks inferred from both healthy and disease cohorts are also of interest, since they can be inferred as being important in healthy microbiomes, but likely changing their roles during dysbiosis, perhaps by an introduction of a pathogenic strain or by triggering one of its virulence factors. For example, the known pathogen *R. torques* has a reduction in its causal influence value between the healthy and diseased cohorts (Fig. 7)^46^.

The *Disease* networks are a novel way of combining the information from the UC and healthy cohorts. The first obvious difference between the network for only UC data (Fig. 5) and the disease network for UC using a combination of UC and healthy data (Fig. 9) is the number of edges—the disease network has more edges than the network without the disease node. It is unclear why more dependencies between the taxa appear in the presence of disease node. One possible explanation is that due to the greater diversity in the samples, which now contains two very different cohorts, there are more dependencies among the variables. Unlike network from only UC data, there are no isolated nodes in the disease network.

More detailed analysis of the UC disease network revealed additional useful information. The taxa, *S. wadsworthensis* (SUTWA) and *B. xylanisolvens* (BACXY) are among the most influential bacteria based on causal effect values (on the special *disease* node) as shown in Table 3. Both taxa are directly connected by an edge to the disease node and have no other directed paths leading to the disease node. The taxon, *E. eligens* (EUBEL), a known beneficial bacterial taxon, has a directed edge to *disease* and directed paths to some other key players such as *B. xylanisolvens* (BACXY) and *S. wadsworthensis* (SUTWA) shown in the supplementary (see Figure S2). We investigated one of the outgoing edges to *F. prausnitzii* and we found from the existing knowledge-bases that both *E. eligens* and *F. prausnitzii* are associated with the metabolite Pectin and the metabolic activity “macromolecular degradation”^65,66^. *R. torques* (RUMTO) is a known pathogenic taxon and has a directed path to the special disease node. *R. torques* is also connected to *R. inulinivorans* (ROSIN1) by an edge. Interestingly *R. torques* is an acetate producer and *R. inulinivorans* is an acetate consumer^67,68^, suggesting a possible mode of causal interaction between the two taxa. *Oscillibacter* is considered an important beneficial taxon, and in the UC disease network it is directly connected to the disease node. It also has multiple paths to disease node via other known beneficial taxa *S. wadsworthensis* and *B. xylanisolvens*, suggesting other unknown modes of interaction contributing to disease.

The analysis of Y-structures identified 18 Y-leg edges. Based on information from the existing knowledge-bases, we discuss the biological significance of the *Y-leg* edge from *Bacteroides fragilis* to *Roseburia intestinalis*. It has been shown that *Bacteroides fragilis* is responsible for producing the metabolite, *acetate*, which accounts for 30–54% of the total products by bacteria^69^. Furthermore, *acetate* is efficiently utilized by certain groups of anaerobic bacteria particularly by butyrate-producing species including *Roseburia intestinalis*^70^. While we can never categorically prove that a Y-leg edge is not counfounded by any hidden factor, we may be able to explain why the edge is significant. The Y-leg edge from *B. dorie* to *Parabacteroides distasonis* is potentially significant because of the intermediate metabolite, Xylan^54,65^.

The methods described in this paper have also been applied to the Crohn’s disease data set. Results can be found in the Supplemental section. We included the causal network inferred from the data collected from the CD cohort (Supplementary Figure S3) and the *causal effects* and *causal influence* values computed from the network.

We discuss a few limitations of the work presented here. The first limitation is that the work presented here assumes that there are no hidden confounders, when in reality we cannot rule out their existence. Minimizing the effects of hidden confounders or measuring unbiased effects in the presence of hidden confounders remains a challenging research direction. Second, the causal influence notion allows us to study the influence of one taxon on the disease node. Future work needs to also consider how groups of taxa influence disease. More generally, future work needs to consider how groups of taxa influence or impact other groups of taxa. A third major limitation is that of *compositionality*, which is caused by the use of *relative abundance* values instead of *raw abundance* values in our analyses. Relative abundance is an attempt to normalize sequencing depth in different samples, but introduce compositionality and the ensuing correlations into the analysis. The *log-ratio* transform and the *hierarchical multinomial-logit* models provide two approaches to address compositionality^71,72^. Unfortunately, the log-ratio method is known to harm the variance strtucture in the data, while the second approach remains to be strongly validated. Finally, future work entails limited laboratory verifications of some of the microbial interactions, especially those involving metabolites.

In summary, this paper takes us one step closer to understanding complex systems such as microbiomes in a causal way. It helps us to shed light on interactions between microbial taxa and the role of metabolites. It provides the framework to include other omics data and understand complex relationships and processes in microbiomes in a quantitative way with the use of interventional calculus. They also make it possible to elucidate biological processes by drawing inferences on the role of intermediaries such as metabolites, genes, and environmental factors. The resulting causal networks are statistically significant, robust, and sensitive. We hypothesize that our approach can lead to a better understanding of the efficacy of probiotics and prebiotics.

## Methods

The first step in inferring causality is to learn the *causal relationships*, which entails discovering the structure of the network of relationships. The next step is to use the structure to infer the *causal effects*, i.e., the magnitude of the strength of causal relationships. Note that the causal network allows us to infer causal effect values even if the nodes are not directly connected by an edge. However, the nodes involved must be connected by a path in order for the causal effect value to be non-zero. The pipeline for causal inference is as follows.

Infer causal networks $\rightarrow$ Apply interventional calculus to compute causal effects $\rightarrow$ Compute causal influence values.

### Problem formulation

To investigate causal relationships in microbiomes, we consider causal networks with nodes corresponding to random variables of interest. The simplest causal network for microbiomes would have nodes representing the relative abundance of every detected microbial taxon, and the edges would represent the causal relationships between the taxa suggesting the direction and magnitude of interactions taking place between the taxa. We will also discuss *disease networks*, a special causal network that has one extra node representing an outcome variable such as the disease status or severity. The edges would either represent the causal relationships between the taxa or between a taxon and the outcome node, highlighting the taxa that are believed to have a direct impact on the outcome along with direction and magnitude of that interaction. More complex microbiome data sets may have nodes representing measurements of different omics entities such as the expression of genes, concentration of metabolites, amount of proteins, methylation data, and more. Additional nodes could also represent host or environmental variables arising from host transcriptome data, host mutational data, host phenotypic data, host clinical data, host medication information, or other environmental conditions that may be measured for the microbiome. An additional level of complexity can be introduced by considering temporal data from longitudinal microbiome studies, which will introduce time-dependant variables of interest. Once a causal network is constructed, interventional calculus can be applied to the resulting network. Used basic probabilistic inference techniques as described by Barber^73^, it is possible to determine the magnitude of the causal impact of one variable of interest on one or more variables of interest.

The goal of the work reported here is to construct causal networks from microbiome data sets, to compute causal effects between all pairs of entities, and to interpret the biological significance of these computations. The causal effects are determined by the regression coefficients under normality assumption. Thus, the magnitude as well as the sign of the causal effect values can be interpreted biologically. The causal network and the resulting computations help us to: (a) identify the key players (most influential taxa) in a microbiome under healthy and disease status, (b) compute the causal effects of individual taxa on the disease outcome. For the first problem, we compute the most *influential* node, which is defined to be the node with the highest *CI* value, where CI is as given in Eq. (8). The *CI* values also help us to compare the impact of the different microbial taxa on the disease node, allowing us to put them in sorted order of influence. In a second problem, we explore causal effects of taxa on the *outcome* or disease node, or vice versa. In general, while the dysbiosis of microbiomes have been strongly associated with disease, it is not known if the dysbiosis is the cause or effect (or both) of the disease. Thus, our techniques allow us to identify taxa most significantly linked to disease or health.

### Data

We worked on both real and simulated data sets. The synthetic data was generated following the logic sampling algorithm^37^. It takes as input three positive integers, *n*, *m* and *d*. It outputs a “true” causal network *G* and a synthetic data set stored as a matrix of size $m \times n$, representing *m* samples each with *n* features or variables of interest that describe the sample. After successfully generating the synthetic data using the above algorithm, we have a *ground truth* causal network model (including “true” network and the “true” regression functions at each vertex) and data generated using such a network model.

As summarized in Table 2, we analyzed the IBD gut microbiome data set by comparing cohorts *A* and *B*. The IBD data set were from the Integrative Human Microbiome Project (iHMP)^39^, and includes data from subjects with Crohn’s Disease (CD), ulcerative colitis (UC), and a cohort of non-IBD (i.e., healthy) subjects that were used as controls.

### Experiments

For each data set (synthetic and real), we generated a causal structure by applying the PC-stable algorithm^28^, after which we computed (a) the causal effect values between every pair of microbial taxa, and (b) the causal influence of each microbial taxon, i.e., the sum total of the (absolute values of) causal effect on all other taxa. For the IBD data set, we also computed the changes in *causal influence* of taxa between diseased and healthy (non-IBD) samples for *iHMP* data. To quantify the causal relationships we applied intervention technique that used linear regression model by ordinary least squares method (under *normality* assumption. We used the coefficients as a measure of the causal effects^27^.

We used the processed data for only the bacterial abundance information downloaded directly from **iHMP** website^39^. The relative abundance matrix was used to generate the causal graphs and then used to estimate the causal effects. The relative abundance is computed by normalizing each raw count with the total number of reads in a sample. In the IBD data set, which included a healthy cohort and diseased cohorts, we also analyzed the data sets by combining the cohorts, but augmenting the causal network with an extra outcome node named *disease* representing the (binary) disease variable. If the severity of the disease were provided, then this node could represent a continuous random variable. This process is called *context embedding*, which is important for causal inference because in different contexts, the same event can be interpreted differently. For the healthy state, the value of disease variable was set to 0, and for the disease state its value was set to 1. We computed the causal effect of all taxa on the disease variable. Note that, in general, while the association may be well established, we do not know if the microbiome composition is the cause or the effect of the disease.

## Supplementary Information

Supplementary Information 1.

## Data Availability

Data used for this study are publicly available by “NIH Integrative Human Microbiome Project”. We used inflamatory bowel disease (IBD) data that includes both Crohn’s Disease (CD) and ulcerative colitis (UC) from “The Inflammatory Bowel Disease Multi’omics Database”. Data repository and download instructions are available at: https://ibdmdb.org/tunnel/public/summary.html. We downloaded taxonomic_profiles.tsv.gz file from metagenomes data type and HMP2_metabolomics.csv.gz file from metabolites data type for further processing and analysis.
