# Supplementary material for: Causal effects in microbiomes using interventional calculus
Source: Sci Rep. 2021 Mar 11;11:5724. doi: 10.1038/s41598-021-84905-3 (PMC7970971; doi:10.1038/s41598-021-84905-3)
Supplement: Supplementary file 1 — Supplementary Information 1. [file 41598_2021_84905_MOESM1_ESM.pdf]

Title: Causal Effects in Microbiomes Using Interventional Calculus

Authors: Musfiqur Sazal, Vitalii Stebliankin, Kalai Mathee, Changwon Yoo, Giri Narasimhan*

Supplemental Section

Log-likelihood : -15071.78

A. muciniphila	AKKMU
A. onderdonkii	ALION
A. putredinis	ALIPU
B. caccae	BACCA
B. cellulosilyticus	BACCE
B. dorei	BACDO
B. eggerthii	BACEG
B. finegoldii	BACFI
B. fragilis	BACFR
B. massiliensis	BACMA
B. ovatus	BACOV
B. sp_2_1_22	BACS2
B. sp_4_3_47FAA	BACS4
B. stercoris	BACST
B. thetaiotaomicron	BACTH
B. uniformis	BACUN
B. vulgatus	BACVU
B. xylanisolvens	BACXY
B. intestinihominis	BARIN
D. invisus	DIAIN
E. coli	ECOLI
E. eligens	EUBEL
E. rectale	EUBRE
E. siraeum	EUBSI
F. prausnitzii	FAEPR
O. unclassified	OSCUN
P. distasonis	PARDI
P. merdae	PARME
P. unclassified	PARUN
P. copri	PRECO
R. intestinalis	ROSIN
R. inulinivorans	ROSIN1
R. torques	RUMTO
S. unclassified	SUBUN
S. wadsworthensis	SUTWA

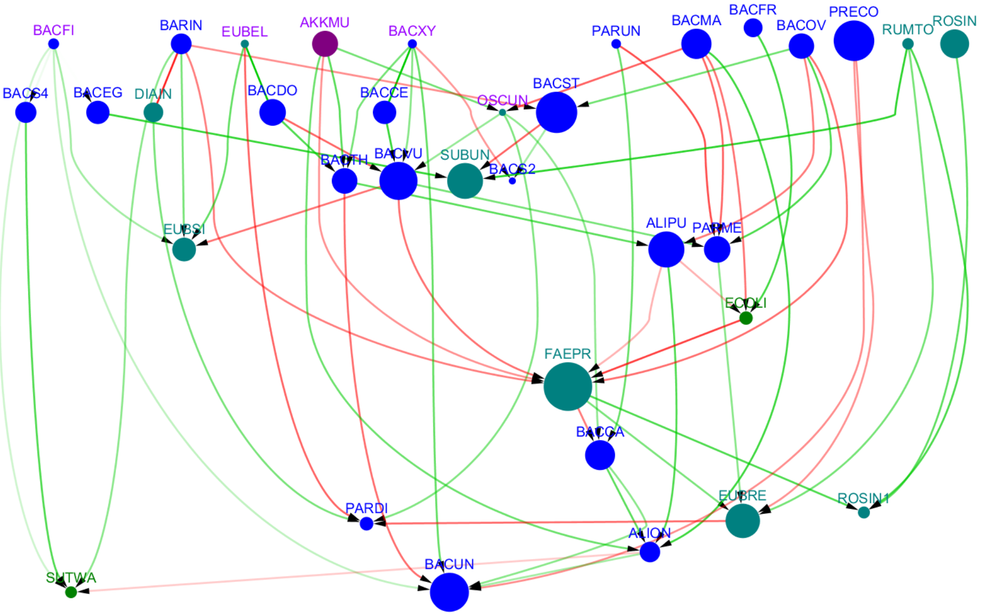

Figure S1. Causal network for non-IBD (healthy) data from iHMP data set. The labels of beneficial bacteria are in purple font. This figure was generated using the R package, `bnlearn`[1], and visualized using `Cytoscape 3.5.0`[2].

A brief discussion on causal analysis of data from CD cohort

Crohn's disease (CD) is another form of IBD that may potentially affect parts of the digestive system. Unlike UC, the links between microbiomes and CD are less established and may be linked to host genetic factors[3]. However, some bacteria are suspected as either beneficial or harmful for subjects with CD. As with the UC data, we calculated the causal influence from the microbiome data for the CD cohort.

Based on the causal influence values, the top-ranked taxa were *R. inulinivorans*, *R. torques*, *B. intestinihominis*, *B. thetaiotaomicron*, *Bacteroides xylanisolvens*, showing considerable overlap with the taxa for UC, possibly even using the same metabolites, genes and pathways. Of these, *R. torques* and *B. thetaiotaomicron* are known to have a harmful impact on the gut of CD subjects[4,5]. On the other hand, *E. eligens* is a beneficial microbe as it helps to grow certain strains of *F. prausnitzii*, a well-known beneficial taxon in CD[6]. *B. xylanisolvens* is known to possess probiotic qualities. Furthermore, it is unable to attach to the gut, which prevents its colonization in the intestines. It is not known to carry plasmid material in the genome of the cell, making it a safer probiotic option. All of these qualities make *B. xylanisolvens* a promising probiotic option[7]. Our approach using causal influence values suggest which bacterial taxa have strong probiotic or harmful potential.

A brief discussion on causal analysis of *Disease* (CD-healthy) network

Similar to UC, we created a disease network using microbiome data from the CD and healthy (non-IBD) cohorts. As before, a sample from the CD cohort would have the value of the disease variable set to 1, while a sample from the healthy cohort would have that value set to 0. Then we measured causal effects of each taxa on the special disease node, which we will refer to as the disease effect. According to absolute values of disease effects, the top taxa are *R. inulinivorans*, *B. finegoldii*, *E. eligens*, *E. siraeum*, *B. caccae*, and *R. torques*.

While *R. inulinivorans* is not known to be directly causally linked to CD, evidence suggests that *R. inulinivorans* is more abundant in CD patients[8]. The taxon *B. finegoldii* is present in the fecal microbiome. While its exact role may be unclear,

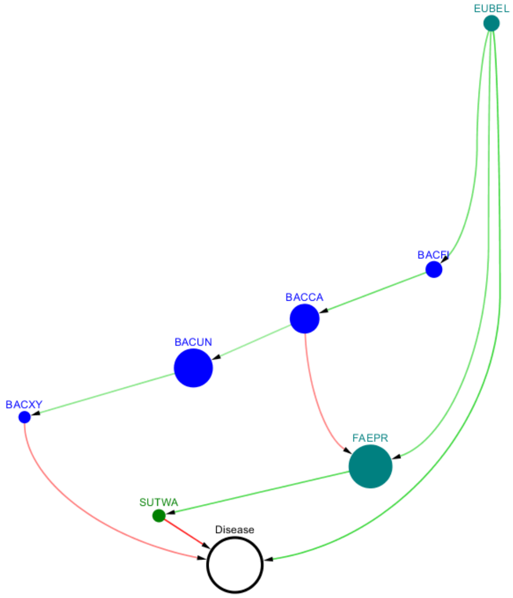

Figure S2. Sub-network of taxa reachable through directed path from *E. eligens* in the *disease* network for Ulcerative Colitis as shown in figure 9 in the paper. This figure was generated using the R package, `bnlearn`[1], and visualized using `Cytoscape 3.5.0`[2].

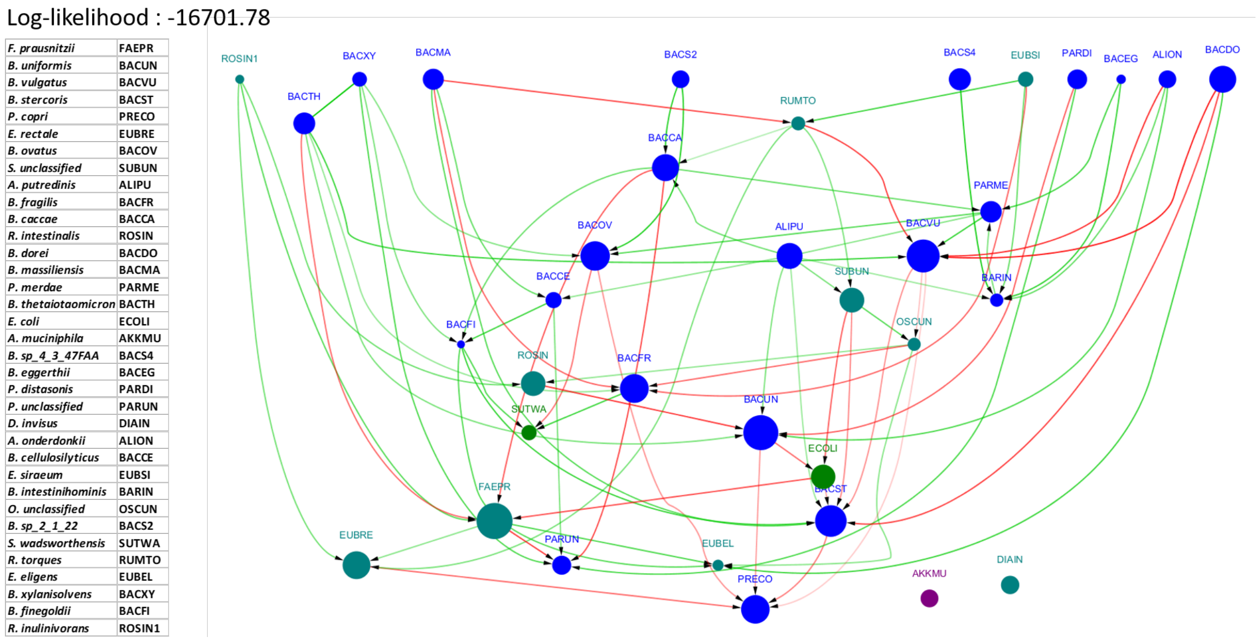

Figure S3. Causal network inferred from Crohn's disease (CD) data set of iHMP database. This figure was generated using the R package, `bnlearn`[1], and visualized using `Cytoscape 3.5.0`[2].

emerging reaserch suggests that it may play a role in maintaining a healthy colon, as well as potentially acting as a probiotic to help patients recover from illnesses such as inflammatory bowel disease and *Clostridium difficile* infection[9]. *E. eligens* is known for its beneficial behavior as a prebiotic in IBD[6]. *B. caccae* can be harmful in some cases for CD and IBD[10] and *R. torques* is a pathogenic taxon for both UC and CD[4]. Thus similar to disease (UC) network analysis we found that the bacterial taxa with the highest causal effect on the disease node are primarily from the beneficial or pathogenic group.

References

1. Scutari, M. Bayesian network constraint-based structure learning algorithms: Parallel and optimised implementations in the `bnlearn` R package. *arXiv preprint arXiv:1406.7648* (2014).
2. Shannon, P. *et al.* Cytoscape: a software environment for integrated models of biomolecular interaction networks. *Genome research* **13**, 2498–2504 (2003).
3. Gevers, D. *et al.* The treatment-naive microbiome in new-onset Crohn's disease. *Cell host & microbe* **15**, 382–392 (2014).
4. Mishra, S. & Imlay, J. A. An anaerobic bacterium, *Bacteroides thetaiotaomicron*, uses a consortium of enzymes to scavenge hydrogen peroxide. *Mol. microbiology* **90**, 1356–1371 (2013).
5. Ricanek, P. *et al.* Gut bacterial profile in patients newly diagnosed with treatment-naive Crohn's disease. *Clin. experimental gastroenterology* **5**, 173 (2012).
6. Chung, W. S. F. *et al.* Prebiotic potential of pectin and pectic oligosaccharides to promote anti-inflammatory commensal bacteria in the human colon. *FEMS microbiology ecology* **93**, fix127 (2017).
7. Chassard, C., Delmas, E., Lawson, P. A. & Bernalier-Donadille, A. *Bacteroides xylanisolvens* sp. nov., a xylan-degrading bacterium isolated from human faeces. *Int. journal systematic evolutionary microbiology* **58**, 1008–1013 (2008).
8. Ananthakrishnan, A. N. *et al.* Gut microbiome function predicts response to anti-integrin biologic therapy in inflammatory bowel diseases. *Cell host & microbe* **21**, 603–610 (2017).
9. Microbewiki. *Bacteroides finegoldii*. (Date last accessed 30-June-2020).
10. Wei, B. *et al.* Molecular cloning of a *Bacteroides caccae* TonB-linked outer membrane protein identified by an inflammatory bowel disease marker antibody. *Infect. immunity* **69**, 6044–6054 (2001).
